# Anti-Cancer Properties of Flaxseed Proteome

**DOI:** 10.3390/proteomes11040037

**Published:** 2023-11-16

**Authors:** Yulia Merkher, Elizaveta Kontareva, Anastasia Alexandrova, Rajesha Javaraiah, Margarita Pustovalova, Sergey Leonov

**Affiliations:** 1 School of Biological and Medical Physics, Moscow Institute of Physics and Technology, Dolgoprudny 141700, Moscow Region, Russialeonov.sv@mipt.ru (S.L.); 2 Faculty of Biomedical Engineering, Technion–Israel Institute of Technology, Haifa 3200003, Israel; 3 Department of Biochemistry, Yuvaraja’s College, University of Mysore Mysuru, Karnataka 570005, India; 4 State Research Center-Burnasyan Federal Medical Biophysical Center of Federal Medical Biological Agency (SRC-FMBC), Moscow 123098, Russia; 5 Institute of Cell Biophysics, Russian Academy of Sciences, Pushchino 142290, Moscow Region, Russia

**Keywords:** metastasis, flaxseed proteins, cancer treatment, radiotherapy, mechanobiology, proteoform level analysis, flaxseed amino-acids

## Abstract

Flaxseed has been recognized as a valuable source of nutrients and bioactive compounds, including proteins that possess various health benefits. In recent years, studies have shown that flaxseed proteins, including albumins, globulins, glutelin, and prolamins, possess anti-cancer properties. These properties are attributed to their ability to inhibit cancer cell proliferation, induce apoptosis, and interfere with cancer cell signaling pathways, ultimately leading to the inhibition of metastasis. Moreover, flaxseed proteins have been reported to modulate cancer cell mechanobiology, leading to changes in cell behavior and reduced cancer cell migration and invasion. This review provides an overview of the anti-cancer properties of flaxseed proteins, with a focus on their potential use in cancer treatment. Additionally, it highlights the need for further research to fully establish the potential of flaxseed proteins in cancer therapy.

## 1. Introduction

Cancer is the second leading cause of mortality worldwide. The majority of cancer-related deaths (90%) are attributed to metastasis, making its reduction a critical factor in improving patients’ survival rates [1]. Recently, there has been a growing proposition that a population of cancer stem cells or cancer initiator cells are responsible for generating highly capable metastatic cells [2,3,4]. Identifying and targeting specific cell populations with the ability to metastasize is absolutely crucial and of the utmost importance, regardless of the mechanisms involved. This will pave the way for more effective and tailored therapeutics. As metastases from primary tumors often occur in sites that are not easily amenable to surgery, such as bones and the brain, chemotherapy remains the primary therapeutic option. However, conventional chemotherapy is associated with significant side effects due to its impact on all fast-proliferating cells.

Metastatic cancers are characterized by the presence of diverse cell populations with varying metastatic abilities and organ specificity. This suggests that the specific molecular characteristics of the metastasizing cells play a crucial role in the metastatic process [5,6,7]. While not all tumors are metastatic, understanding the unique properties of these cells is essential for comprehending their metastatic potential. Nevertheless, the development of proficient metastatic cells continues to be a subject of debate. The process of metastasis is commonly described as a carefully choreographed series of events, where the microenvironment plays a critical role in determining the ultimate outcome [8,9,10]. The tumor’s capability to generate secondary metastatic lesions is commonly known as the tumor’s “metastatic potential” (MP).

One broadly utilized indicator of cancer’s propensity to metastasize is cell invasiveness. During the invasion of the surrounding tissues, cancer cells are able to rapidly change their shape and apply forces to facilitate the invasion process. Cells with high metastatic potential (MP) are typically more receptive to internalizing materials from their surroundings, displaying a more dynamic intercellular composition than benign or low MP cells. The cytoskeleton of highly metastatic cells is denser [11,12,13]. While common chemotherapeutics primarily target cancer cell proliferation and growth, addressing metastatic spread requires more intricate and innovative approaches, such as radiotherapy. Anti-metastatic drugs aim to inhibit processes like vascularization, microenvironment-dependent cell growth, cell binding to the extracellular matrix (ECM), and stem-like properties [14,15]. In clinical cases where metastasis is expected, an aggressive treatment strategy combining tumor growth and metastasis targeting is typically employed. Unfortunately, these aggressive treatments often compromise patients’ quality of life and increase the risk of kidney or liver-related toxicity [16], and in some cases, inappropriate treatment might even elevate the chance of metastasis development. Thus, accurate assessment of metastatic risk, followed by the identification of specific personalized treatments that can potentially reduce or inhibit metastasis formation, holds paramount importance.

Treatment with flaxseed proteins may induce changes in cytoskeleton dynamics that influence a cell’s ability to change shape and apply force, affecting its invasive capacity. During penetration through the extracellular matrix (ECM) and endothelial transmigration (intravasation/extravasation), invading cancer cells must alter their morphology based on the local microenvironment conditions. Leading cells often display pseudopods and filament-based protrusions, aiding them in navigating through narrow pores [17,18]. The mechanobiological response to chemotherapy, coupled with changes in cell viability, dictates the treatment’s success and overall effectiveness [19]. 

According to reports from the World Health Organization (WHO) [20,21], unhealthy eating habits and lack of physical activity contribute to about 30% of cancer-related deaths. Approximately one-third of diagnosed cancers could be prevented or mitigated by promoting physical activity and adopting healthier dietary habits. Various studies, including those conducted by the European Medicines Agency, support the potential benefits of plants used in traditional cancer medicines. Flax, a globally significant agricultural crop, is abundant in bioactive molecules that may possess anti-cancer properties. The biological activity of dietary proteins often leads to the development of bioactive peptides released during digestion in the gastrointestinal tract. These peptides can enter the bloodstream by crossing the digestive epithelial barrier, enabling them to reach distant organs and provide beneficial effects for the organism [22].

## 2. Flaxseed Proteins

Generally, whole flaxseed comprises 30–41% fat, 20–35% dietary fiber, 20–30% protein, 4–8% moisture, 3–4% ash, and 1% simple sugars [23]. Recently, researchers have begun exploring the anti-cancer properties of flaxseed proteins, uncovering various ways in which they may exhibit such properties. For instance, flaxseed stands out as a significant dietary source of lignans, a type of phytoestrogen recognized for its anti-cancer attributes [24]. Lignans have been found to inhibit the growth of hormone-sensitive cancers, such as breast and prostate cancers, by interfering with the effects of estrogen on these tissues [25]. Additionally, flaxseed offers an abundant supply of alpha-linolenic acid—an omega-3 fatty acid. Omega-3 fatty acids are known for their anti-inflammatory effects, potentially reducing cancer risk [26,27]. Moreover, there is speculation that omega-3 fatty acids could hinder or limit the proliferation and dissemination of certain types of cancer cells [28]. The rich antioxidant content in flaxseed contributes to cellular protection against damage induced by free radicals [29,30]. Free radicals, unstable molecules, can induce cellular damage and escalate cancer risk. Certain studies have even indicated that flaxseed proteins might possess anti-angiogenic properties, impeding the formation of new blood vessels essential for tumor growth [21].

**Figure 1:**
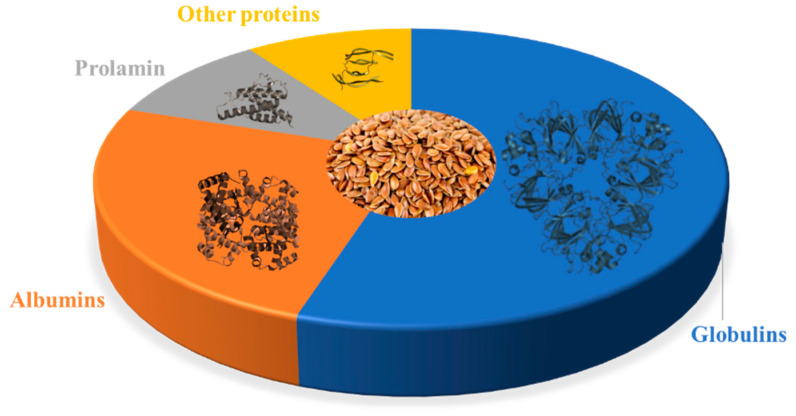
Flaxseed protein composition. Note: percentages may vary depending on the source and processing of the flaxseed.

The composition of flaxseed proteins can fluctuate based on factors such as flax variety, growth conditions, and processing techniques. However, on average, flaxseed proteins comprise roughly 15–25% albumins, 43–80% globulins, 4–10% prolamins, and up to 10% other proteins [31,32,33,34,35,36,37,38], as illustrated in Figure 1.

The level and composition of flaxseed proteome depends on many factors, for example, cultivars, environmental conditions, and processing methods [37]. Flaxseed proteins mainly consist of 11S globulin and 2S albumin (Table 1). The 11S globulin is a salt-soluble protein; it has high molecular weight (252–298 kDa). The 2S albumin is a water-soluble protein; it has low molecular weight (16–17 kDa) [33,37,39]. 

## 3. Albumins of Flaxseed

Albumin, a type of protein present in various foods such as egg whites and whey protein, is recognized for its water-soluble nature and high digestibility, making it a valuable source of essential amino acids. While flaxseed albumin has not been as extensively studied as other components like lignans or omega-3 fatty acids, there exists some evidence to suggest its potential anti-cancer properties. Functioning as a potent antioxidant, albumin aids in neutralizing free radicals—unstable molecules responsible for cellular damage and heightened cancer risk [29]. Furthermore, albumin plays a pivotal role in immune system functionality, safeguarding against endothelial dysfunction through immunomodulation and antioxidant mechanisms [56]. Diminished albumin levels could potentially trigger inflammation and an increase in leukocyte count [57]. As a significant source of amino acids, albumin contributes to essential protein synthesis required for healthy cell growth and division; deficiencies in this synthesis have been correlated with an elevated cancer risk [58,59]. Albumin is also responsible for transporting vital nutrients, including vitamins and minerals, throughout the body [60], which is crucial for maintaining a robust immune system and minimizing cancer risk. Beyond these roles, albumin can serve as a carrier for chemotherapy drugs, enhancing their targeted delivery to tumor sites and potentially lowering metastasis risk [61].

The extracellular matrix (ECM), a complex network of proteins and molecules surrounding cells, plays a pivotal role in cancer progression and metastasis. Evidence indicates that albumin interacts with and regulates ECM components, potentially inhibiting cancer cell invasion and metastasis. Albumin’s interactions encompass key proteins involved in cancer cell invasion, like matrix metalloproteinases (MMPs) and urokinase-type plasminogen activator (uPA) [62,63]. Moreover, albumin can engage with other ECM proteins, such as laminin and fibronectin, contributing to the inhibition of cancer cell invasion [64,65,66].

The primary form of albumin in flaxseed is 2S albumin, a seed storage protein. Although the precise physiological and metabolic role of 2S albumins remains to be definitively described, evidence based on their amino acid composition and mobilization during germination suggests their function as nitrogen and sulfur donors [67]. Typically existing as heterodimers, these proteins consist of 8–16 kDa water-soluble polypeptides [67], connected by two disulfide bonds resistant to pepsin and trypsin. While the subunit compositions and structures of 2S albumins differ, their 3D form is generally a compact sphere enriched in α-helices [68,69]. An extensively studied anti-cancer peptide derived from plants is lunasin, a small peptide from the 2S albumin family, containing 43–44 amino acid residues [45]. Lunasin encompasses multiple functional domains, including an aspartic acid tail, an RGD domain, and a chromatin-binding helical domain [70]. Lunasin exhibits a wide-ranging therapeutic effect against cancer in both laboratory and live models, encompassing lung cancer, colon cancer, leukemia, melanoma, and breast cancer [71,72]. This protein holds the potential to hinder cancer cell invasion and migration (Table 2) and represents a promising avenue for further research in developing novel cancer treatments.

Overall, while further research is required to comprehensively comprehend the potential anti-cancer effects of 2S albumin, this protein shows promise in potentially inhibiting cancer cell invasion and migration. Thus, it stands as a promising realm of investigation for the advancement of novel cancer treatments.

## 4. Globulins of Flaxseed

Globulins form a diverse family of proteins present in various foods, including legumes, nuts, and animal products. Among flaxseed proteins, globulin takes precedence as the principal component, its size being reported to be in the range of 252–298 kDa (for 11–12 S Globulins). Comprising about 3% α-helical and 17% β-structures [83,84,85], globulins, akin to albumins, have not undergone extensive examination for their anti-cancer attributes. Nevertheless, some evidence indicates the potential anti-cancer properties of globulins.

Globulins play a pivotal role in the immune system, actively combating infections and diseases, including cancer. There has been a suggestion that globulins might stimulate white blood cell production, contributing to the battle against cancer cells [86]. Certain globulins have been shown to impede the activity of enzymes linked to tumor growth and metastasis. For instance, soybean globulins have been observed to hinder the action of tyrosine kinase, an enzyme central to cancer cell proliferation [87,88]. Certain globulins, like whey protein, boast significant antioxidant content that aids in neutralizing free radicals and guarding against cellular damage that could lead to cancer [89]. Moreover, globulins partake in regulating hormone levels within the body [90,91,92]. Given that hormones can influence the development and progression of specific cancers, modulating their levels might yield anti-cancer effects as well.

Predominantly, flaxseed’s major globulin type is the 11S globulin (comprising over 85% of all globulins), while the 7S (less than 2%) and 2S Vicilin-like globulins (less than 4%) are considered minor [93]. The non-reduced 11S globulin reveals five polypeptide bands, including a basic subunit (18–20 kDa), an acidic subunit (30–40 kDa), and additional polypeptides with molecular weights of 47, 80, 120, and 160 kDa. Studies exploring the distinct biological properties of flaxseed protein digests have yielded varied results, contingent on the methods employed. For example, the highest antioxidant activity (90%) and effective fungal inhibition were observed with glutelin hydrolysates, whereas intact glutelin protein exhibited the highest angiotensin-converting enzyme-inhibiting (60%) activity [34]. There is some evidence suggesting that 11S globulin (also referred to as glutelin [94]), a major storage protein in flaxseed, might possess anti-cancer properties, potentially inhibiting cancer cell proliferation and inducing apoptosis (Table 3). However, it is crucial to acknowledge that these investigations were conducted in vitro (within a laboratory setting using cell cultures), and further research is necessary to ascertain the potential effects of glutelin in vivo (within living organisms).

## 5. The Proteoform Level Analysis of Major Proteins from Flaxseed

While studies on 2S albumin have focused on its characterization, structural properties, allergenicity, and functional aspects, detailed proteoform-level analysis has been somewhat limited in comparison. Nevertheless, certain studies underscore the significance of proteoform-level analysis in comprehending the structural diversity, post-translational modifications (PTMs), and functional attributes of 11S globulins sourced from different plants. Over the past two decades, mass spectrometer methods have been employed and refined to identify the types and locations of these modifications in purified proteins. To examine certain types of PTMs, including phosphorylation, acetylation, methylation, glycosylation, ubiquitination, and sumoylation—considered the most prevalent and well-studied—a set of specialized mass spectrometric techniques has been developed. These techniques systematically scan peptides derived from a protein to identify the presence of specific modifications [102].

Typically, following protein hydrolysis into peptides and amino acids, the samples can undergo pre-fractionation, achieved through techniques like chromatography, ion-exchange chromatography, or gel electrophoresis. This process divides the hydrolysate into specific subpopulations of compartments, such as post-translational modifications (PTMs), which can be enriched using various methods like affinity resins and specific antibodies. Subsequently, these fractions are individually analyzed using reversed-phase liquid chromatography coupled with mass spectrometry (LC–MS), where selected peptides undergo tandem mass spectrometry (MS/MS). Alternatively, electrospray ionization mass spectrometry (ESI–MS) and electrospray ionization tandem mass spectrometry (ESI–MS/MS) can be employed. Following this analysis, the fraction is collected and loaded into a silica gel column for reversed-phase high-performance liquid chromatography (HPLC) coupled with electrospray ionization time-of-flight mass spectrometry (ESI–TOF-MS), and the compound is further analyzed using nuclear magnetic resonance (NMR) [32,103]. For instance, research delving into proteoform-level analysis of soybean 11S globulin, referred to as glycinin [104], and peanut 11S globulin, known as arachin, was conducted using mass spectrometry-based proteomics [105]. For glycinin, various proteoforms exhibiting distinct PTMs—like phosphorylation, acetylation, methylation, and disulfide bond formation—were identified through two-dimensional gel electrophoresis (2DE) and mass spectrometry [106]. Similarly, multiple proteoforms of lupin 11S globulin, characterized by varying isoelectric points (pI) and molecular weights, were detected [107]. These lupin 11S globulin proteoforms displayed differences in solubility, digestion patterns, and IgE-binding capacity, hinting at potential implications in terms of allergenicity [108].

## 6. Extraction and Characterization of Flaxseed’s Amino Acids

**Figure 2:**
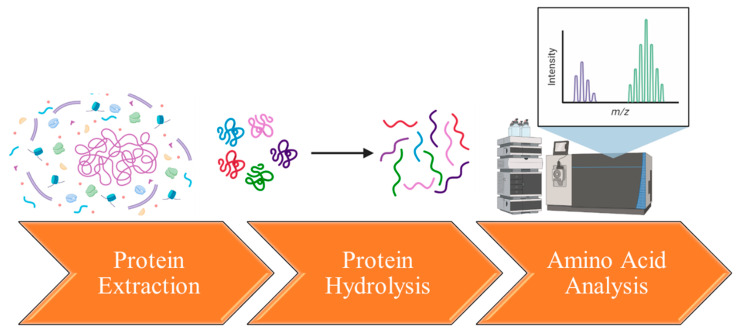
Isolating and characterizing amino acids from flaxseed proteins (created with BioRender.com, accessed on 1 July 2023).

The isolation and characterization of amino acids from flaxseed proteins generally involve a series of steps, including protein extraction, hydrolysis, and analysis (Figure 2). Flaxseed proteins are initially extracted from the seeds using appropriate extraction buffers or solvents. Common methods encompass saline extraction, phosphate buffer extraction, or sodium dodecyl sulfate (SDS) extraction [109,110,111]. These methods aid in solubilizing and separating proteins from other seed components. Following successful extraction, hydrolysis is conducted to disintegrate the proteins into individual amino acids. Two frequently employed techniques are acid hydrolysis and enzymatic hydrolysis. Acid hydrolysis involves treating the protein sample with controlled conditions using hydrochloric acid (HCl) or sulfuric acid (H_2_SO_4_) [108]. Acid hydrolysis offers a deeper level of protein cleavage and eliminates the risk of bacterial contamination in the hydrolysate. However, this method comes with drawbacks, including the complete degradation of tryptophan, methionine, cystine, and cysteine, as well as the partial breakdown of oxy-amino acids (serine and threonine). Furthermore, acid hydrolysis leads to the deamination of amide bonds in asparagine and glutamine, resulting in the formation of ammonia nitrogen and aspartic and glutamic acids. It also causes the destruction of vitamins and the formation of challenging-to-separate humic substances [112]. An additional challenge is the production of substantial amounts of salts, especially chlorides and sulfates, known for their toxicity to the body. As a result, acid hydrolysates necessitate further purification, typically employing ion-exchange chromatography in the production process. Consequently, the technology of acid hydrolysis proves to be labor-intensive, demanding complex equipment and additional purification stages for the resultant drugs [113].

On the other hand, enzymatic hydrolysis employs proteolytic enzymes like trypsin, chymotrypsin, or pepsin to cleave proteins into smaller peptide fragments and, eventually, amino acids [114,115,116]. Enzymatic hydrolysis offers the advantage of being conducted under mild and easily maintained conditions. Amino acids are virtually preserved, avoiding additional reactions like racemization. Enzymes, being more specific, provide better control over the process. The hydrolysis of proteins can be accomplished through a single enzymatic step or a sequential enzyme hydrolysis involving multiple enzymes. The selection of enzymes depends on the protein source and the specific requirements of the end user [117]. Enzymatic hydrolysis of proteins offers the capability to generate short-chain bioactive peptides with diverse biological properties, including a reduction in protein allergenicity [118]. For instance, when flaxseed protein undergoes enzymatic hydrolysis with alcalase, it yields a significant quantity of free antioxidant amino acids—such as histidine, lysine, tyrosine, methionine, and tryptophan. The resulting low-molecular-weight peptides exhibit notable antioxidant and regenerative abilities in the experiment [119].

The hydrolyzed amino acids are subsequently quantified and characterized using diverse analytical methods. The primary technique is high-performance liquid chromatography (HPLC) coupled with UV detection or fluorescence detection [120]. Amino acids are separated based on their chemical properties, such as polarity, using a suitable chromatographic column and elution gradient. The resulting peaks corresponding to individual amino acids are identified and quantified with the aid of calibration curves created using amino acid standards. To verify the identity of the amino acids, mass spectrometry (MS) can be employed [106]. 

Gathering both quantitative and qualitative insights into the amino acid composition of flaxseed proteins holds considerable value in comprehending their nutritional quality, functional attributes, and potential applications in cancer treatment. It is important to acknowledge that the amino acid makeup within flaxseed proteins can vary based on the specific flaxseed variety and the analytical methods employed for amino acid analysis (Table 4).

In the realm of cancer therapy, specific amino acids can serve as supplements or be incorporated into treatment strategies to bolster efficacy and alleviate treatment-related side effects. Notably, glutamic acid, a predominant amino acid found in flaxseed proteins, exhibits anticancer properties and has even been proposed as an anticancer agent [121]. Due to its adept penetration capability, high compatibility, and low immunogenicity, glutamic acid has been regarded as a drug nano-delivery platform [122]. A combination of aspartic and glutamic acids has been shown to inhibit tumor cell proliferation, induce tumor cell death, and hold substantial promise for clinical utilization as an anti-cancer agent [123]. Modifying the widely accepted anti-tumor treatment with peptides containing arginine, glycine, and aspartic acid has demonstrated a noteworthy antiproliferative impact [124]. Arginine, known for its immunostimulatory effect [125], has been extensively explored as a cell-penetrating agent for drug delivery in the form of arginine-containing peptides [126,127]. Additionally, isoleucine, when administered at high doses, has been observed to suppress the proliferation of breast and lung cancer cells [128]. While leucine is not typically employed as a direct cancer treatment, it assumes pivotal roles in diverse metabolic processes and might bear implications for cancer metabolism and treatment strategies.

**Table 4:** Major amino-acids of flaxseed proteins are aliphatic, acidic, non-essential, crystalline α-amino acids. The schematic structure prepared using © Karl Harrison 3DChem.com (accessed on 1 July 2023).

Amino Acid	Function	Structure	Composition from Flaxseed Proteins
Glutamic Acid	neurotransmitter, contributes to the synthesis of proteins, plays a role in energy production	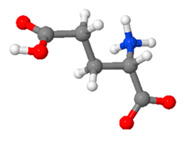 C_5_H_9_NO_4_	19–27% [129,130,131]
Aspartic Acid	building block for other amino acids and nucleotides, contributes to energy production, participates in the urea cycle	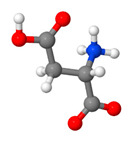 C_4_H_7_NO_4_	8–21% [132,133,134]
Arginine	micronutrient, stimulate the immune system, increase production of nitric oxide, participate in detoxification of nitrogenous wastes	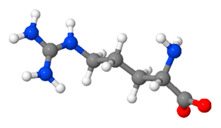 C_6_H_14_N_4_O_2_	8–12% [135,136,137]
Isoleucine	regulation of hemoglobin synthesis, detoxification of nitrogenous wastes, stimulation of immune function	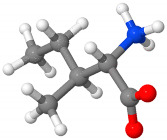 C_6_H_13_NO_2_	4–8% [133,138,139]
Leucine	important for protein synthesis and hormone production, contributes to regulation of blood-sugar levels, growth and repair of muscle and bone	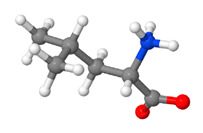 C_6_H_13_NO_2_	4–7% [131,132,134,136]

## 7. Presence of Flaxseed Proteome in Databases

Despite the evident significance of flaxseed proteins as potential anti-cancer agents, their comprehensive study and description remain somewhat lacking when compared to proteins from other plant sources. For instance, *Linum usitatissimum* (Flax) has a total of 936 entries within the UniProt database, with merely 11 of these entries reviewed by the Swiss-Prot database. In contrast, *Glycine max* (Soybean), another prominent seed source, boasts a staggering 84,712 entries in the UniProt database, with 431 of these entries reviewed by Swiss-Prot. Additionally, while the proteomes of soybeans have been extensively described and documented in protein databases, the proteomes of flax are notably absent from these databases.

Among the few flax proteins covered by UniProt databases and reviewed by Swiss-Prot are those involved in diverse functions such as lignan biosynthesis, disease resistance against pathogens, enzymatic activities in the catabolism of cyanogenic glycosides, protease inhibition, and more. These proteins play roles in biosynthesis of secondary metabolites (59%), conferring resistance to pathogens (33%), and are rarely employed in biotechnological applications (8%).

The existing NLM database mainly highlights protein families like Glycosyltransferase 2, DEAD box helicase, LTN1, SCAMP, RNA polymerase alpha/beta chains, ATPase alpha/beta chains, and Peroxidase, among others. However, limited information is available about the core components of the flaxseed proteome (Table 1), especially when compared to the diversity of protein coverage for various other plants (Table 5). 

While specialized databases like the Plant Proteome Database (developed by ©Klaas J. van Wijk Lab, Cornell University) exist for plants such as Arabidopsis, maize, and rice, there is currently no such database dedicated to flax. Individual databases have been established for specific plant species including Arabidopsis (TAIR database), maize (maizeGDB), tomatoes (TOMATOMICS), rice (Rice genome annotation project database), soybeans (SoyBase), and wheat (Wheat proteome database) [140], but a dedicated database for flax is notably absent. 

A thorough and comprehensive examination of the primary proteins in flaxseed is essential. This involves enhancing existing databases with additional information on flaxseed proteins. Furthermore, creating a dedicated database specifically for flax proteins will contribute to a deeper understanding of the properties of the flaxseed proteome, ultimately strengthening its potential for cancer treatments.

## 8. The Sequencing and Profiling of Flaxseed Proteins

Protein sequencing is a pivotal process that involves determining the exact sequence of amino acids constituting a protein. This step is indispensable for comprehending the protein’s structure, function, and potential applications. However, the sequences and comprehensive profiles of flaxseed proteins have yet to be documented in databases. Nonetheless, significant advancements have been made in sequencing the main proteins of flaxseed, such as 2S-albumin and 11S-globulin, in comparison to their counterparts from other plant sources.

**Figure 3:**
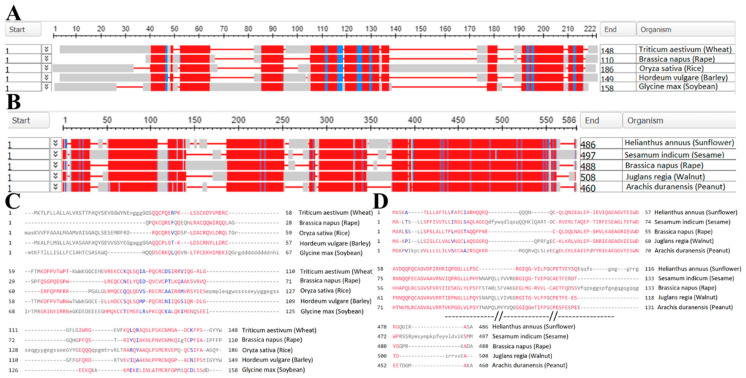
The main flaxseed proteins’ profiling and sequencing. Sequence comparison and alignment was performed using Basic Local Alignment Search Tool (BLAST). (**A**,**B**), respectively—alignment of 2S albumins profiles and 11S globulins profiles from various plant sources. Alignment columns with no gaps are colored in blue or red. The red color indicates highly conserved columns and blue indicates less conserved ones. (**C**,**D**)—sequences of 2S albumins and 11S globulins, respectively. The red color indicates conserved residues, blue indicates residues with no gaps, gray uppercase indicates that less than 50% of the sequences contain gaps, gray lowercase – more than 50% of the sequences contain gaps.

Figure 3 demonstrates a remarkable degree of similarity in protein profiles across various plant sources. This suggests that computational models could potentially be employed to predict the typical structures and sequences of the primary flaxseed proteins. While some progress has been made in profiling and sequencing flaxseed proteins, it is worth noting that only 47 proteins have been identified using the BLAST database, with sequence coverage ranging from 6% to 49% [141].

Further exploration and comprehensive profiling of flaxseed proteins, including a focus on post-translational modifications, will undoubtedly contribute to a better understanding of protein functionality, stability, and their potential applications in anti-cancer strategies.

## 9. The Combined Anti-Cancer Action of Flaxseed Proteins

Flaxseed proteins can be consumed as part of the diet and subsequently absorbed within the digestive tract. Once absorbed, they can enter the bloodstream and circulate throughout the body, potentially reaching cancer cells. There is also the possibility of delivering flaxseed proteins directly to cancer cells through targeted drug delivery systems or nanoparticle formulations. Moreover, the topical application of flaxseed oil, which contains certain proteins present in flaxseed, has been explored as a potential method for delivering these proteins to skin cancer cells [142,143].

The efficacy of flaxseed proteins in various cancer cell lines, such as breast [144], skin [71], prostate, and colon [74] cancer cells, has been demonstrated. Flaxseed proteins have exhibited anti-tumor activity by inducing cell cycle arrest and promoting apoptosis (programmed cell death) [32]. Notably, flaxseed proteins possess the capacity to modulate the immune system, which holds a pivotal role in cancer surveillance and elimination [142]. Furthermore, flaxseed proteins have been observed to enhance the effectiveness of chemotherapy drugs across different cancer cell lines [27,145].

According to the ClinicalTrials.gov database, there are no past or current clinical trials specifically focused on cancer treatment using proteins from flaxseed. However, 21 clinical trials have been conducted to explore the biological effects of dietary flaxseed, either alone or in combination with chemotherapy or radiotherapy, on cancer patients (only 3 of them—with results and none of them on phase 4). Unfortunately, no significant positive results or in-depth investigations into the underlying mechanisms of action have been recorded. For instance, there were no notable effects of flaxseed on aromatase inhibitors’ activity concerning selected breast tumor characteristics or serum steroid hormone levels [146]. In patients with locally advanced or metastatic non-small cell lung cancer (NSCLC) undergoing radiotherapy, the daily intake of flaxseed led to noteworthy treatment-related gastrointestinal toxicities, even though there was a low reported incidence of acute radiation-induced complications [147]. On the other hand, there is an indication that a dietary milled sesame/pumpkin/flax seed mixture, added to a habitual diet, lowered triglyceride and CRP, TNF-alpha, and IL-6 levels, affected glycemic control, and improved fatty acid profile and pruritus symptoms [148]. Currently (2023), there are only two ongoing clinical trials investigating the use of flaxseed in cancer treatment.

**Figure 4:**
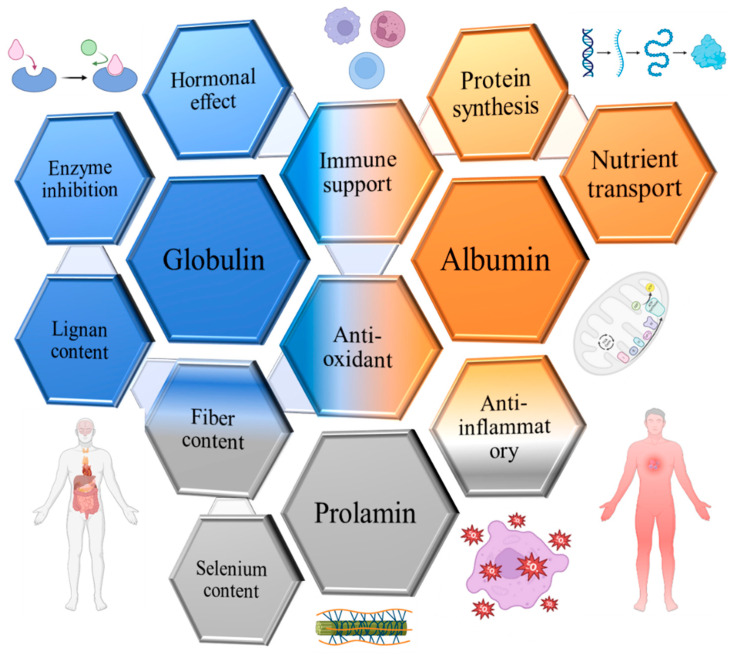
Anti-cancer action of main flaxseed proteins.

In essence, the potential advantages of utilizing extracted and especially selected flaxseed proteins in cancer treatment are indeed promising (Figure 4). However, further research is imperative to fully elucidate their underlying mechanisms of action and gauge their clinical effectiveness in treating cancer.

## 10. Flaxseed Proteome Effect on Anti-Cancer Radiotherapy

Radiotherapy (RT) stands as a prominent method for cancer treatment, functioning by inducing DNA damage within cancer cells. This complex medical procedure necessitates meticulous planning and monitoring. However, despite advancements in RT techniques, certain tumors display notable radio resistance, leading to elevated rates of treatment failure and tumor relapse [149,150]. Upon encountering DNA damage, cancer cells elevate their antioxidant activity [151] and metabolic processes, encompassing glucose flux, amino acid metabolism, and fatty acid utilization. This metabolic response provides the necessary substrates and energy for the repair of DNA damage [152].

Although research specifically focusing on the impact of flaxseed proteins on radiotherapy remains limited, it is crucial to recognize that flaxseed proteins could potentially offer benefits during radiotherapy due to their antioxidant and anti-inflammatory properties. Flaxseed 2S-albumin, akin to other albumin proteins, possesses antioxidant attributes (Table 1 and Table 2). Antioxidants play a role in neutralizing the free radicals generated during radiotherapy [153], thereby reducing oxidative stress and potential harm to healthy tissues. Through its scavenging action against free radicals, flaxseed 2S-albumin might confer protective effects on cells exposed to radiation.

Some studies propose that specific globulin proteins exhibit anti-inflammatory characteristics (Table 1 and Table 3). Inflammation is a natural reaction to radiation exposure [154], but excessive or prolonged inflammation could contribute to tissue damage. By lessening inflammation, flaxseed 11S-globulin might potentially alleviate some of the detrimental effects of radiotherapy on healthy tissues.

Flaxseed proteins, including 2S-albumin and 11S-globulin, furnish essential amino acids and contribute to nutritional support for the body. Maintaining sufficient protein intake is vital during radiotherapy to facilitate tissue repair and overall well-being [155]. Flaxseed proteins can play a role in meeting the nutritional requirements of individuals undergoing radiotherapy, potentially supporting recovery and minimizing treatment-related complications.

Previous research has demonstrated that the intake of flaxseed oil increased the survival rate of rats exposed to 8 Gy gamma radiation. However, this effect was hypothesized to stem from indirect biological mechanisms, such as impacts on the gut microbiota, rather than the direct radioprotective qualities of flaxseed oil [156]. For instance, after being metabolized by the intestinal microbiota, flax components could modify mammary gland miRNAs, potentially reducing the risk of adult breast cancer [157]. Flaxseed has also shown potential in mitigating side effects induced by radiotherapy. In mice subjected to a combination of thoracic radiation therapy and a flaxseed diet, a decrease in p53-responsive miR-34a was observed. This miRNA is associated with cellular maturity and apoptosis regulation [158]. Mice fed with flaxseed exhibited reduced expression of lung injury biomarkers (Bax, p21, and TGF-beta1), oxidative lung damage, lung fibrosis, and inflammatory cell influx into lungs following 13.5 Gy thoracic X-ray radiation exposure. However, the radioprotective effect of flaxseed on Lewis Lung carcinoma was not observed [159,160]. Consequently, targeting tumor metabolism through the flaxseed proteome presents a promising therapeutic avenue for safeguarding normal cells during radiation while sensitizing cancer cells to its effects.

## 11. Further Research

Delivering proteins to cancer cells is a complex field of research, and the effectiveness of different delivery methods can vary depending on the specific protein and type of cancer cells. More investigation is necessary to optimize the delivery of flaxseed proteins to cancer cells. Nanoparticles, for instance, can be tailored to encapsulate and deliver proteins to specific cells or tissues, including cancer cells. Functionalized nanoparticles can enhance cellular uptake and intracellular delivery of proteins. The successful delivery of anticancer proteins using biocompatible nanoparticles has been previously demonstrated [161].

Liposomal delivery is another approach worth considering. Liposomes are lipid-based vesicles capable of encapsulating proteins for targeted delivery. They can be engineered to improve stability, cellular uptake, and controlled release of proteins at the intended site. For example, encapsulating drugs into liposomes enables the creation of injectable medications with prolonged effects. A key advantage of liposomes is their capacity to accumulate in the tumor zone. Additionally, these liposomes interact with plasma proteins, forming complexes that are subsequently captured by macrophages from the bloodstream. To ensure intravenously administered drugs effectively reach target cells in the body, various methods are proposed to reduce opsonization. This includes incorporating lipids with a high phase transition temperature into the liposomal membrane or producing vesicles with a size not exceeding 100 nm [162]. However, the most efficient approach involves modifying the liposome surface with glycolipids, such as monosialogangliosides, phosphatidylinositol, cerebroside sulfate, and lipid derivatives of polyethylene glycol (PEG). These modified liposomes, known as stealth liposomes, boast a half-life of up to 24 h in mice and rats and up to two days in the human body. This extended half-life contributes to the selective accumulation of active substances in tumors [163]. Liposomal delivery systems have been explored for various therapeutic proteins, including those with anticancer properties [164]. Drugs based on stealth liposomes–Caelix (Schering-Plough Europe, Brussels, Belgium) and Doxil (ALZA, Mountain View, CA, USA), AmBisome (Gilead Sciences, Foster City, CA, USA) and Amphocil (Liposome Technology, Menlo Park, CA, USA)—have been clinically tested and approved by the FDA [163]. While specific studies involving flaxseed proteins are scarce, liposomal delivery methods can potentially be adapted for their delivery.

An alternative approach for protein delivery involves the use of dendrimers. Dendrimers belong to a distinctive class of polymers characterized by a highly branched structure. Notably, their size and shape can be precisely defined during chemical synthesis. Target molecules can bind to dendrimers either by forming complexes with their surface or by embedding themselves deeply between individual chains. The controlled dimensions and surface properties, coupled with the stability of dendrimers, make them potentially viable as carriers for protein delivery [165]. It is also worth considering carbon nanoparticles, specifically nanotubes and fullerenes. These particles exhibit an enhanced affinity for lipid structures and can establish stable complexes with peptides and DNA oligonucleotides, even encapsulating these molecules. This characteristic makes them valuable in the development of efficient delivery systems for vaccines and genetic material [166].

Furthermore, proteins can be modified or engineered to enhance their stability, cellular uptake, and affinity for cancer cells. For example, fusion with targeting peptides or antibodies specific to cancer cell surface receptors can facilitate the selective binding and internalization of proteins into cancer cells. This approach has been successfully employed for the targeted delivery of diverse therapeutic proteins [61]. An increasingly prevalent approach involves utilizing hybrid structures where the protein vector and the target substance are covalently linked. The selectivity of the conjugate’s action is achieved either through the presence of specific receptors on the surface of tumor cells, recognizable by a vector protein or antibody, or due to a significantly higher level of vector protein receptors on the surface of tumor cells compared to normal cells. Several studies have published data showcasing the successful utilization of cytotoxic conjugates created based on cytotoxic antibiotics and vector molecules. These conjugates effectively deliver the antibiotic to tumor cells, demonstrating promising results in targeted therapy [167]. Identifying crucial proteoforms is of the utmost importance as they contribute to the development of potential anti-cancer treatments. Highlighting the significance of proteoform-level analyses is crucial for advancing research in this direction most effectively.

**Figure 5:**
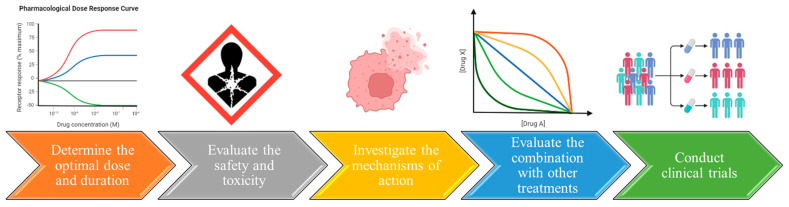
Further research is required in order to establish cancer treatment with flaxseed proteins (created with BioRender.com, accessed on 1 July 2023).

Finally, in order to establish cancer treatment with flaxseed proteins, more research is needed (Figure 5) to: (a) Investigate the mechanisms of action–unveiling the specific mechanisms through which individual flaxseed proteins inhibit cancer cell growth and metastasis is vital. This understanding will aid in developing more targeted and effective therapeutic strategies; (b) Determine the optimal dose and duration of treatment. Determining the appropriate dose and treatment duration is critical for the efficacy of flaxseed protein treatment against cancer cells. Tailoring the dosage and duration for different cancer types is essential; (c) Evaluate the potential of flaxseed proteins in combination with other treatments–exploring the potential synergies between flaxseed proteins and other anti-cancer treatments, like chemotherapy and radiation therapy, could enhance treatment outcomes while minimizing side effects; (d) Evaluate the safety and toxicity of flaxseed protein; despite the promising anti-cancer effects observed, the safety and toxicity profiles of flaxseed proteins in humans need thorough evaluation through clinical trials; and (e) Conduct clinical trials: conducting well-designed clinical trials is paramount to assessing the efficacy and safety of flaxseed proteins in cancer treatment. Larger sample sizes, longer treatment periods, and placebo-controlled designs are imperative for obtaining reliable and robust results.

## 12. Conclusions

Nutrition plays a crucial role in the overall well-being of cancer patients and can significantly contribute to reducing complications during treatment [168,169]. In recent years, there has been a growing focus on plant-based nutrition as a means to protect against a range of leading causes of death, including various types of cancer such as breast, prostate, colorectal, and gastrointestinal cancers. Plant-based diets comprising whole foods have demonstrated substantial protective effects against these cancers and other chronic diseases. These diets can serve as disease-modifying agents, enhancing the management and treatment of these conditions. Consequently, interventions involving nutrition in the prevention of diverse cancers offer a notable complement to existing medical therapies.

Addressing the current gaps in knowledge about flaxseed proteins is crucial for advancing both cancer treatment and overall health. Firstly, the limited representation of flaxseed proteins in existing databases underscores the need for a more comprehensive inclusion of this vital information. Additionally, the absence of established sequences for flaxseed proteins poses a challenge in understanding their biological activities. Furthermore, the scarcity of research on the properties of flaxseed for cancer treatment emphasizes the importance of exploring these avenues. The development of a compatible nutraceutical enriched with flaxseed proteins and the analysis of its anti-metastatic effects contribute significantly to promoting healthy aging. These initiatives not only address existing gaps but also pave the way for novel strategies that can enhance both cancer treatment and overall well-being.

## Figures and Tables

**Table 1:** Proteome components found in flaxseed, and their potential heals benefits. Images prepared using Mol* Viewer 1.1, RCSB Protein Data Bank software (RCSB PDB, Piscataway, NJ, USA).

Proteome Component	Structure	Percentage of Total Protein	Potential Health Benefits
11S Globulin	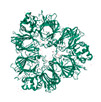	43–48%	Contains anti-cancer peptides [40,41,42]; may have anti-inflammatory and antioxidant effects [43,44]
2S Albumin	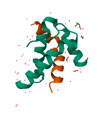	16–18%	Contains peptides with potential anti-cancer and anti-inflammatory effects [42,45,46]; may have cholesterol-lowering effects [47]
7S Vicilin-Like Globulin	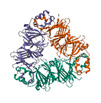	11–13%	May have antioxidant and anti-inflammatory effects [44,48]; contains peptides with potential anti-cancer effects [49]
Prolamin	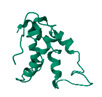	4–6%	Contains peptides with potential anti-cancer effects [50,51]
Glutelin-Like Protein	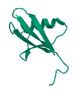	2–3%	May have antioxidant and anti-inflammatory effects [52,53]
Other Proteins	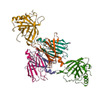	10–15%	May include lignans, which have antioxidant and anti-inflammatory and thus potential anti-cancer effects [54,55]

**Table 2:** Anti-metastatic action of 2S albumin component–Lunasin.

Action	Mode of Action	Evidence
Inhibits cancer proliferation and induce apoptosis	Interaction with the αvβ3 integrin via the FAK/ERK/NF-κB signaling pathway; activation of caspase-3 and cleavage of PARP [73,74]	Inhibited the proliferation and the tumorsphere-forming capacity of **colon cancer** HCT-116 cells [75]. Increased the amount of **colon** cancer cells KM12L4 undergoing apoptosis twofold [76]. Reduced tumor growth in **NSCLC** and melanoma xenografts [72].
Inhibits cancer cell invasion and migration	Interaction with cellular receptors (integrins and EGFR); disrupts the activity of key signaling proteins (MMP-2/-9) via the FAK/Akt/ERK and NF-κB signaling pathways [77]	Caused a reduction in the migration (scratch assay) of HCT-116 and KM12L4 **colon cancer** cells [74]. Inhibited the migration and invasion activity in MDA-MB-231 and MCF-7 **breast cancer** cell lines [77]. Inhibited the invasion of **melanoma** A375 and B16-F10 cells to matrigel [71].
Reduces inflammation and oxidation	Reduction in the production of certain inflammatory markers (ROS, TNF-α and IL-6) [78,79]	Mouse **macrophage cells** (LPS-stimulated RAW 264.7) reduced the production of certain inflammatory markers (ROS, TNF-α and IL-6) [80].
Reduces cancer cells colonization	Reduction in phosphorylation of the intracellular kinases FAK and AKT; reduction in histone acetylation of lysine residues in H3 and H4 histones [71]	Reduced pulmonary colonization after injection of highly metastatic B16-F10 **melanoma** cells [71,72]. Inhibited formation of **liver** metastasis in murine model [74].
Arrest cell cycle	Arrests the cell cycle at the G2/M and G1/S phase; altered the expression of the G1 specific cyclin-dependent kinase complex components, increased levels of p27Kip1, reduced levels of phosphorylated Akt [76,81]	Inhibited cell cycle progression at the G1/S phase for **NSCLC** H661 cells [81]. Lunasin treatment of MDA-MB-231 **breast cancer** cells resulted in a notable increase in RB1 level, which lead to arrest of G1 phase [82]

**Table 3:** Anti-tumor action of 11S globulin components.

Action	Mode of Action	Evidence
Inhibits cancer cell growth and proliferation	Lectin was responsible for the antiproliferative activity of the MPI-h [95]. Acid fraction of glycinin, composed of low molecular weight peptides, was able to inhibit cancer cells growth [96].	Inhibited the proliferation of UMR106 rat **osteosarcoma**-derived cells [95] and inhibited the growth of **cervical cancer** HeLa cells in a dose-dependent manner [96].
Antioxidant effect	Small (~1 kDa) peptides generated from 11S globulin inhibit the formation of hydroxyl radicals by reaction of H_2_O_2_ and Co^+2^ and decreasing ROS [41]	Demonstrated peroxyl radical scavenging activity dependent on the structure of peptides in human **adenocarcinoma** cell line, Caco-2 TC7 [41]
Induces cancer cell death (apoptosis)	Increased the expression levels of apoptosis-related spot-like protein (ASC), caspase-1, the cleaved gasdermin D, and interleukin-1β [97]. Flaxseed orbitides influence mitochondrial- and death receptor-mediated intrinsic and extrinsic pathways [98].	Glutelin extracts induced the apoptosis of **cervical cancer** HeLa cells [99]. Human **gastric** SGC-7901 cell apoptosis was induced by linusorb B3 flaxseed orbitides [98]
Arresting cell cycle	Linoorbitides arrested the cell at the G1 phase by downregulation of CDK2/4 and overexpression of p21WAF1/CIP1 and p27KIP1 genes [100].	Cyclolinopeptides/linusorbs are capable of arresting cell cycle, and thus reduce metastasis spreading in human **gastric** SGC-7901 cells [100].
Cytotoxic effect on cancer cells	Linoorbitides have strong cytotoxic effect probably due to its binding abilities to human serum albumin [32].	Linusorb B3 is cytotoxic to human **melanoma** cells A375 and **breast cancer** cells Sk-Br-3 and MCF7 at high concentration [101].

**Table 5:** Coverage of main flaxseed proteins from plant sources in current databases. Protein’s synonyms are listed below the table.

	Proteins	11S Globulin	2S Albumin	7S Vicilin-like Protein	Prolamin
Plants					
*Linum usitatissimum* (Flax)		0	12	0	0
*Triticum aestivum* (Wheat)		3397	3944	22	2925
*Brassica napus* (Rape)		136	2252	57	998
*Oryza sativa* (Rice)		73	1920	20	215
*Hordeum vulgare* (Barley)		286	539	2	284
*Glycine max* (Soybean)		19	574	70	62
*Arachis hypogaea* (Peanut)		15	342	13	29
*Avena sativa* (Oat)		182	210	0	174
*Helianthus annuus* (Sunflower)		34	181	2	8
*Sesamum indicum* (Sesame)		9	105	13	4
*Juglans regia* (Walnut)		8	102	4	3
*Carya illinoinensis* (Pecan)		4	92	5	18

**11S Globulin**–Cruciferin; Glycinin; 12S seed storage globulin; 13S globulin seed storage protein; Legumin; Glutelin; Glutelin-like protein; Gliadin; Glutenin; **2S Albumin**–2S seed storage albumin protein; Conglutin; Conlinin; Glutenin; Gliadin; Puroindoline; Phytepsin; Hordoindoline; Hordein; Avenin; Vromindoline; Avenoindoline; Tryptophanin; Napin; Non-specific lipid-transfer protein; **7S Vicilin-like protein**–Basic 7S globulin; Vicilin; Cupincin; Globulin-1 S; Beta-conglycinin; Leginsulin; PA1b; Plant functional proteins/peptides; γ-Conglutin; Albumin-1; **Prolamin**–Hordoindoline; Hordein; Gliadin; Avenin; Avenoindoline; Egg cell-secreted protein; Puroindoline; Serpin; Vromindoline; Tryptophanin.

## Data Availability

Publicly available datasets were analyzed in this study. This data can be found here: RCSB.org (accessed on 1 November 2023), 3DChem.com (accessed on 1 July 2023), Uniprot.org (accessed on 1 November 2023), NCBI.nlm.nih.gov (accessed on 1 November 2023), PPDB.tc.cornell.edu (accessed on 1 August 2023), Arabidopsis.org (accessed on 1 August 2023), Rice.uga.edu (accessed on 1 August 2023), Gramene.org (accessed on 1 August 2023), Maizegdb.org (accessed on 1 August 2023), Soybase.org (accessed on 1 August 2023), Clinicaltrials.gov (accessed on 1 November 2023).
